# An immune cell infiltration-based immune score model predicts prognosis and chemotherapy effects in breast cancer

**DOI:** 10.7150/thno.49451

**Published:** 2020-10-25

**Authors:** Silei Sui, Xin An, Caiming Xu, Zongjuan Li, Yijun Hua, Geya Huang, Sibei Sui, Qian Long, Yanxia Sui, Yuqing Xiong, Micheal Ntim, Wei Guo, Miao Chen, Wuguo Deng, Xiangsheng Xiao, Man Li

**Affiliations:** 1 The Second Affiliated Hospital of Dalian Medical University; Institute of Cancer Stem Cell, Dalian Medical University, Dalian, China.; 2 Sun Yat-Sen University Cancer Center, State Key Laboratory of Oncology in South China, Collaborative Innovation Center of Cancer Medicine, Guangzhou, China.; 3 Department of General Surgery, The First Affiliated Hospital of Dalian Medical University, Dalian, China.; 4 Institute (College) of Integrative Medicine, Dalian Medical University, Dalian, China.; 5 The First People's Hospital of Yichang (People's Hospital of Three Gorges University), Yichang, Hubei, China.; 6 Vagelos College of Physicians and Surgeons, Columbia University, New York, USA.; 7 Department of Physiology, Dalian Medical University, Dalian, China.

**Keywords:** tumor-infiltrating immune cells, breast cancer, CIBERSORT, immune score, prognostic

## Abstract

**Background:** Immune cells have essential auxiliary functions and influence clinical outcomes in cancer, with high immune infiltration being associated with improved clinical outcomes and better response to treatment in breast cancer (BC). However, studies to date have not fully considered the tumor-infiltrating immune cell (TIIC) landscape in tumors. This study investigated potential biomarkers based on TIICs to improve prognosis and treatment effect in BC.

**Results:** We enrolled 5112 patients for analysis and used cell type identification by estimating relative subsets of RNA transcripts (CIBERSORT), a new computational algorithm, to quantify 22 TIICs in primary BC. From the results of univariate Cox regression, 12 immune cells were determined to be significantly related to the overall survival (OS) of BC patients. Furthermore, least absolute shrinkage and selection operator (LASSO) and multivariate Cox regression analyses were applied to construct an immune prognostic model based on six potential biomarkers. By dividing patients into low- and high-risk groups, a significant distinction in OS was found in the training cohort, with 20-year survival rates of 42.6% and 26.3%, respectively. Applying a similar protocol to validation and test cohorts, we found that OS was significantly shorter in the high-risk group than in the low-risk group, regardless of the molecular subtype of BC. Using the immune score model to predict the effect of BC patients to chemotherapy, the survival advantage for the low-risk group was evident among those who received chemotherapy, regardless of the chemotherapy regimen. In evaluating the predictive value of the nomogram, a decision curve showed better predictive accuracy than the standard tumor-node-metastasis (TNM) staging system.

**Conclusion:** The immune cell infiltration-based immune score model can be effectively and efficiently used to predict the prognosis of BC patients as well as the effect of chemotherapy.

## Introduction

Tumor progression is a complex process that requires interaction between cancer cells, the microenvironment, and the immune system, influencing both tumor initiation and progression 1. Recent research suggests that immune system cells have an essential accessory role of preserving tissue integrity and function during homeostasis, infection, and noninfectious perturbations by eliminating pathogens, exerting some influence on the clinical outcomes of tumors 2, 3. Many studies have also demonstrated that high immune infiltration is associated with improved clinical outcomes and better response to treatment in breast cancer (BC) 4-11.

Tumor-infiltrating lymphocytes (TILs) comprise a considerable part of tumor-infiltrating immune cells (TIICs). It has been shown that TILs inhibit tumor growth and correlate with improved clinical outcomes in melanoma 12, 13, colorectal cancer 14-16, and ovarian cancer 17, 18. Additionally, higher levels of TILs are associated with better disease-free survival (DFS) and overall survival (OS) in human epidermal growth factor receptor 2-positive (HER2+) and triple-negative primary breast cancer (TNBC) 19, leading to clinical trials of several immunotherapeutic agents in TNBC 20. Moreover, in patients with HER2-positive tumors and TNBC, TILs are also associated with a higher pathological complete response (pCR) rate following neoadjuvant therapy 9, 21.

TIICs also differentiate into tumor-associated macrophages (TAMs) and tumor-infiltrating dendritic cells (TIDCs), which can promote tumor growth and metastasis 22-24. Therefore, it is not surprising that higher levels of TAMs and TIDCs are strongly associated with poor outcomes in BC 24-26. However, as the immune microenvironment is complex and characterized by many immune cell networks, studies have not taken full account of the entire TIIC landscape in tumors. Accordingly, it is imperative to find potential biomarkers based on the complete TIIC landscape to improve prognosis prediction and treatment effect in BC.

Cell type identification by estimating relative subsets of RNA transcripts (CIBERSORT) is a new computational algorithm for enumerating immune cell subsets using bulk gene expression data 27. In this study, we employed CIBERSORT to quantify 22 TIICs in primary BC. Moreover, using least absolute shrinkage and selection operator (LASSO) regression and multivariate regression analysis, we established a novel immune-based model to provide a powerful means for predicting the survival and benefits of chemotherapy in patients with BC. We further validated the prognostic model using 175 BC tumor samples based on RNA sequencing data.

## Results

### Gene expression profile database selection according to enrollment criteria

**Figure 1:**
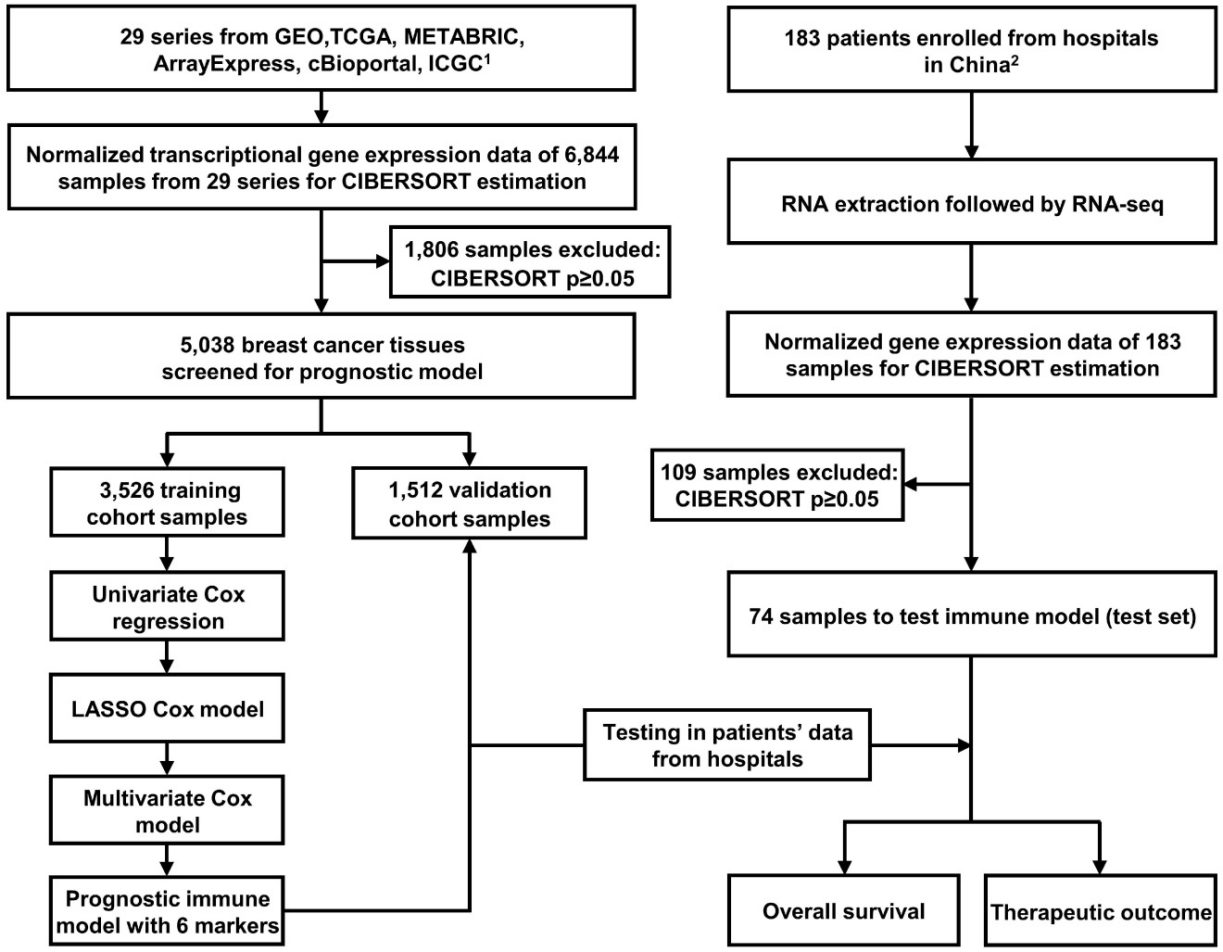
** Flow chart of the study design.** In total, 6,844 breast cancer samples from 29 public data series were used to perform CIBERSORT. Six immune markers were ultimately screened from LM22 to construct a prognostic immune model. The training (N = 3,526) and validation (N = 1,512) cohorts used were derived these public datasets. Another test cohort (N = 74) was from hospitals in China. Part 1. Inclusion criteria were as follows (prognostic model): **(1)** datasets containing more than 50 human primary BC samples; **(2)** series providing overall survival time and survival status; **(3)** transcriptome profiling as the experimental method. Part 2. Enrollment criteria were as follows: **(1)** pathology confirmed as primary BC following surgery; **(2)** complete clinical records and follow-up information available; **(3)** no history of other tumors; **(4)** informed consent.

The study workflow design is depicted in Figure 1. The following databases were selected to obtain gene expression profiles of BC tissues: (1) The Cancer Genome Atlas (TCGA, https://portal.gdc.cancer.gov/), (2) Gene Expression Omnibus (GEO, https://www.ncbi.nlm.nih.gov/geo/), (3) ArrayExpress (https://www.ebi.ac.uk/arrayexpress/), (4) International Cancer Genome Consortium (ICGC, https://icgc.org/), and (5) Molecular Taxonomy of Breast Cancer International Consortium (METABRIC, the data were downloaded from cBioPortal website: http://www.cbioportal.org/). We systematically searched these databases with the term “breast cancer”. The enrollment criteria for the prognostic model were as follows: datasets containing more than 50 human primary BC samples, series presented with OS time and survival status, and transcriptome profiling as the experiment type. As indicated in Figure 1, 29 series (6,844 BC samples in total) were ultimately included for constructing the prognostic model. The studies obtained from each of the databases are summarized together with accession numbers in Table S1.

### Establishment of the prognostic immune score model

**Figure 2:**
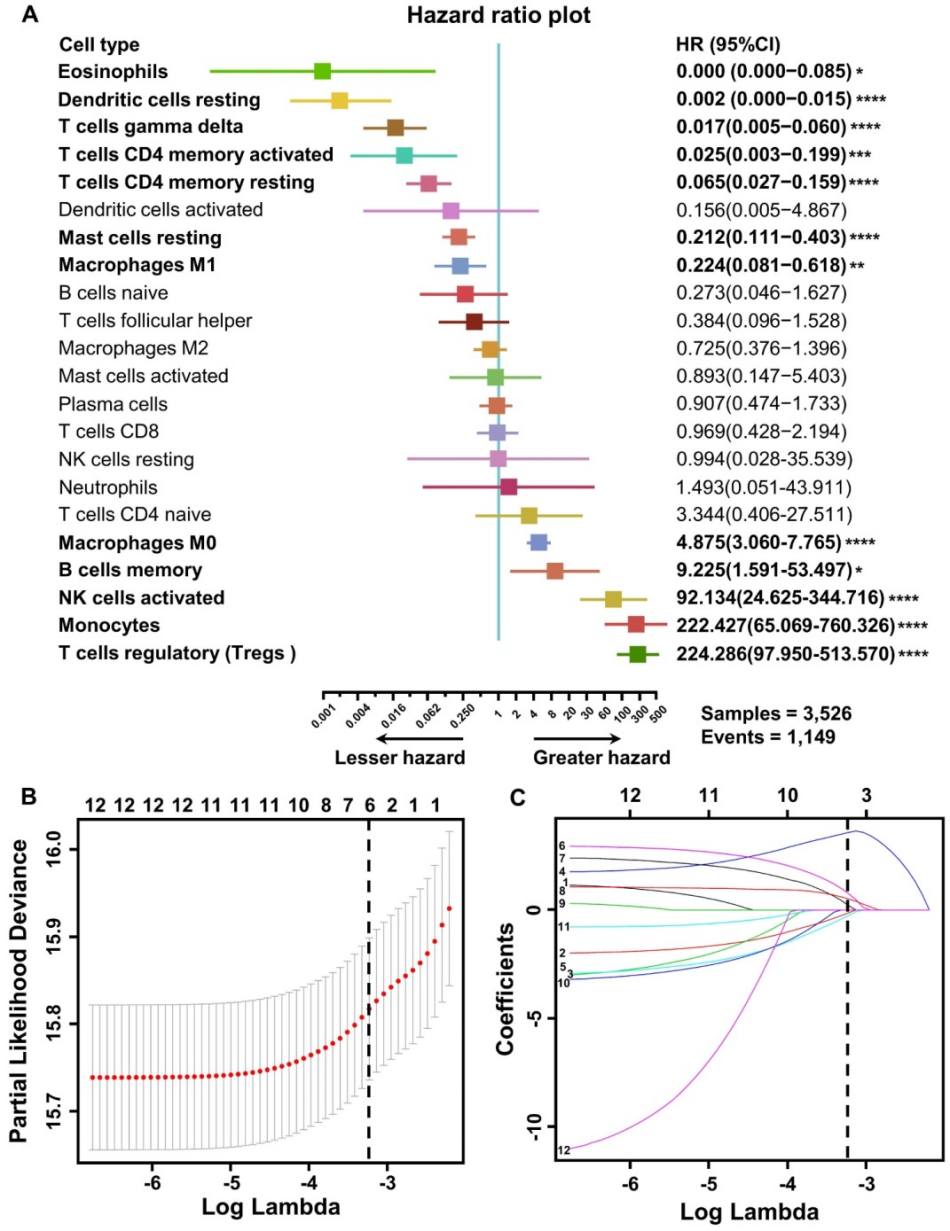
** Construction of the prognostic immune model in the training cohort. (A)** Forest plots of the univariate Cox hazard model for overall survival. Unadjusted HRs are shown with 95% confidence intervals. *p < 0.05, **p < 0.01, ***p < 0.001, ****p < 0.0001. The direction indicating less hazard favors better survival, whereas the direction indicating greater hazard favors lower survival. **(B)** Partial likelihood deviance for LASSO coefficient profiles. The red dots represent the partial likelihood values, the gray lines represent the standard error (SE), and the vertical dotted line is shown at the optimal values by 1 - s.e. **(C)** Least absolute shrinkage and selection operator (LASSO) coefficient profiles of 12 immune cells. Immune cell types: 1. memory B cell; 2. CD4 memory resting T cells; 3. memory activated T cell; 4. regulatory T cell (Treg); 5. gamma delta T cell; 6. activated NK cell; 7. monocyte; 8. M0 macrophage; 9. M1 macrophage; 10. resting dendritic cell; 11. resting mast cell; 12. eosinophil.

**Figure 3:**
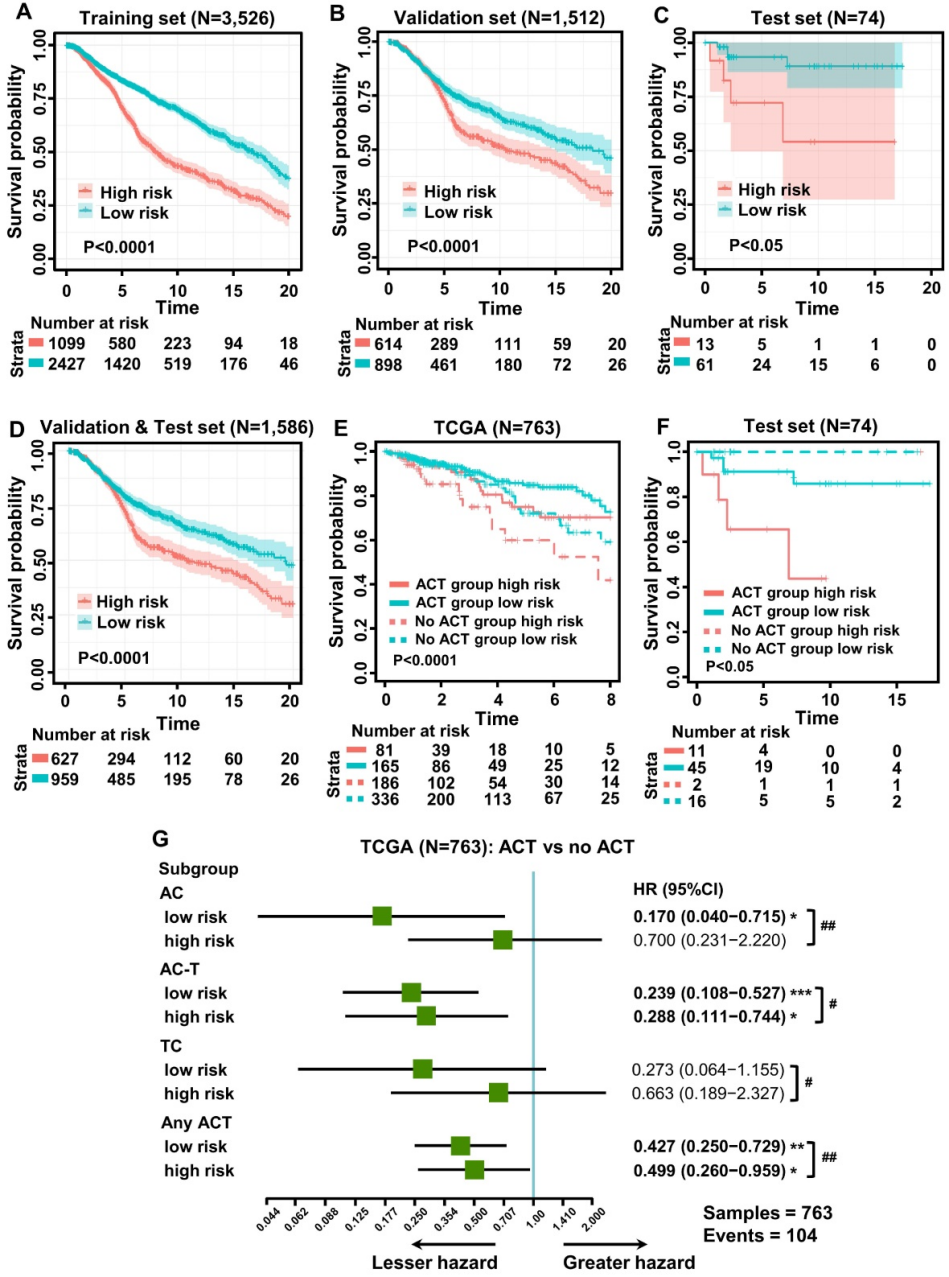
** Kaplan-Meier curves of survival for low- and high-risk patients. (A)** Overall survival (OS) in the training cohort. **(B)** OS in the validation cohort. **(C)** OS in the test cohort. **(D)** OS in the combination of validation and test cohorts. **(E)** Subgroup analysis of adjuvant chemotherapy (ACT) benefit for disease-free survival (DFS) of low- and high-risk patients in the TCGA database. **(F)** Survival analysis of neoadjuvant chemotherapy response (pCR, pathologic complete response; no pCR, including pathologic partial response, pathologic stable disease, and pathologic progression of the disease) among patients with different risk stratifications (test cohort). **(G)** Forest plot showing ACT benefit for DFS of low- and high-risk patients with different chemotherapy regimens in TCGA. Hazard ratios, with 95% confidence intervals, are shown for patients with ACT compared with no ACT treatment in each different risk group. ACT vs no ACT: *p < 0.05, **p < 0.01, ***p < 0.001, ****p < 0.0001. Low risk vs high risk: *^#^p <* 0.05, *^##^p <* 0.01, *^###^p <* 0.001, *^####^p <* 0.0001. AC-T, anthracycline plus cyclophosphamide followed by taxane; AC, anthracycline, and cyclophosphamide; TC, taxane, and cyclophosphamide.

To explore the prognostic value of tumor-infiltrating immune cells, stratified sampling was used to divide 5,038 samples into a training cohort (N = 3,526, Table S2) and a validation cohort (N = 1,512, Table S2) in a ratio of 7:3. Figure 2A shows a forest plot of the associations between each immune cell subset and OS in the training cohort. According to the results of the univariate Cox hazard model, eosinophils (*p =* 0.015), resting dendritic cells (*p <* 0.0001), gamma-delta T cells (*p <* 0.0001), resting and activated CD4+ T cells (*p <* 0.0001; *p =* 0.0005), resting mast cells (*p <* 0.0001), M0 and M1 macrophages (*p <* 0.0001; *p =* 0.004), memory B cells (*p =* 0.013), activated NK cells (*p <* 0.0001), monocytes (*p <* 0.0001), and regulatory T cells (*p <* 0.0001) were significantly related to OS in BC patients. Subsequently, we performed LASSO Cox regression to select highly relevant variables from among the 12 (univariate Cox regression: *p <* 0.05), obtaining results of Lambda.min = 0.00115 [log (Lambda.min) = -6.766], lambda.1se = 0.0395 [log (Lambda.1se) = -3.231] (Figure 2B). Resting CD4+ T cells, regulatory T cells, gamma-delta T cells, activated NK cells, monocytes, and M0 macrophages were included when log (Lambda.1se) = -3.231 (Figure 2C). Multiple Cox regression was performed to further identify independent predictors and calculate the prognostic index. We established the formula for the prognostic immune score model according to multiple Cox regression (Risk score = regulatory T cells * 2.526 - resting CD4 T cells * 1.761 - gamma-delta T cells * 2.334 + activated NK cells * 3.408 + monocytes * 2.645 + M0 macrophages * 1.591) (Table S3), with the immune score of each sample from the training cohort calculated according to this model. Subsequently, all samples from the training cohort were divided into high- or low-risk groups using the cutoff of 0.374 28, as determined by the OptimalCutpoints package in R. To evaluate the OS of these low- and high-risk patients, Kaplan-Meier curves were generated, and a significant distinction was observed in the training cohort (Figure 3A), regardless of the molecular subtype of BC (Figure S1). The 20-year survival rates were 37.8% and 20.0% for the low- and high-risk groups, respectively [hazard ratio (HR): 2.72, 95% confidence interval (95%CI): 2.40-3.08, *p <* 0.0001] (Table 1).

### Validation of the prognostic immune score model

To evaluate the effect of this prognostic model, the same formula and prognostic immune score model were applied to the validation cohort, the test cohort (cases from hospitals in China), and the combination of the validation and test cohorts. Patients from the validation and test cohorts were grouped by the cutoff value calculated from the training set (0.374), and Kaplan-Meier curves were generated for the cohorts. Based on the results, OS was significantly shorter in the high-risk group than in the low-risk group in the validation cohort (Figure 3B), test cohort (Figure 3C), and mixed cohort (Figure 3D), regardless of the molecular type of BC (Figure S2). In addition, the 20-year survival rates were 47.8% and 30.1% for the low- and high-risk groups, respectively, (HR: 2.10, 95%CI: 1.74-2.53, *p <* 0.0001) in the combined validation and test cohorts (Table 1).

### The prognostic immune score model predicted the effect of chemotherapy

As neoadjuvant chemotherapy (neo-ACT), as well as adjuvant chemotherapy (ACT), has been reported to be related to immune infiltration 29, we further evaluated whether the application of chemotherapy (CT) would influence the prognosis of BC. According to the NCCN Guidelines in Oncology (National Comprehensive Cancer Network, Clinical Practice Guidelines in Oncology, Breast Cancer, Version 5, 2020 https://www.nccn.org/professionals/physician_gls/default.aspx), anthracycline + cyclophosphamide (AC), AC followed by taxane (AC-T) and taxane + cyclophosphamide (TC) are major chemotherapy regimens. Information regarding the administration of CT was collected from TCGA and METABRIC datasets and hospitals in China. To evaluate the relationship between the immune score and chemotherapy effect, the same formula was applied to the cohorts from TCGA, the hospitals in China, and METABRIC. The patients of these three cohorts were divided by the cutoff value (0.374) into low- and high-risk groups, and the DFS advantage for the low-risk group was evident in all three cohorts, regardless of whether they received chemotherapy (Figure 3E-F, Figure S3). Detailed information on ACT was documented only for the cohort from TCGA. Compared with patients who did not undergo chemotherapy in the low-risk group, the survival advantage was evident in patients who received AC and AC-T chemotherapy schemes (*p <* 0.05; *p <* 0.001). In contrast, the chemotherapy benefit in the high-risk group was only observed with the AC-T chemotherapy scheme (*p <* 0.05, Figure 3G). More importantly, further evaluation of subgroup interaction showed that low-risk patients obtained better chemotherapy effects, regardless of the chemotherapy regimen (AC: *p <* 0.01; AC-T: *p <* 0.05; TC: *p <* 0.05; Figure 3G). Furthermore, we used data from the test cohort of neo-ACT to assess the association between the immune prognostic model and the effect on chemotherapy. As illustrated in Figure S4, there was a tendency toward a higher immune score in the neo-ACT-sensitive group (pCR status) than in the neo-ACT-resistant group (non-pCR status), though no significant difference was observed (*p* = 0.129).

### The nomogram system improved the prognostic immune score model

**Figure 4:**
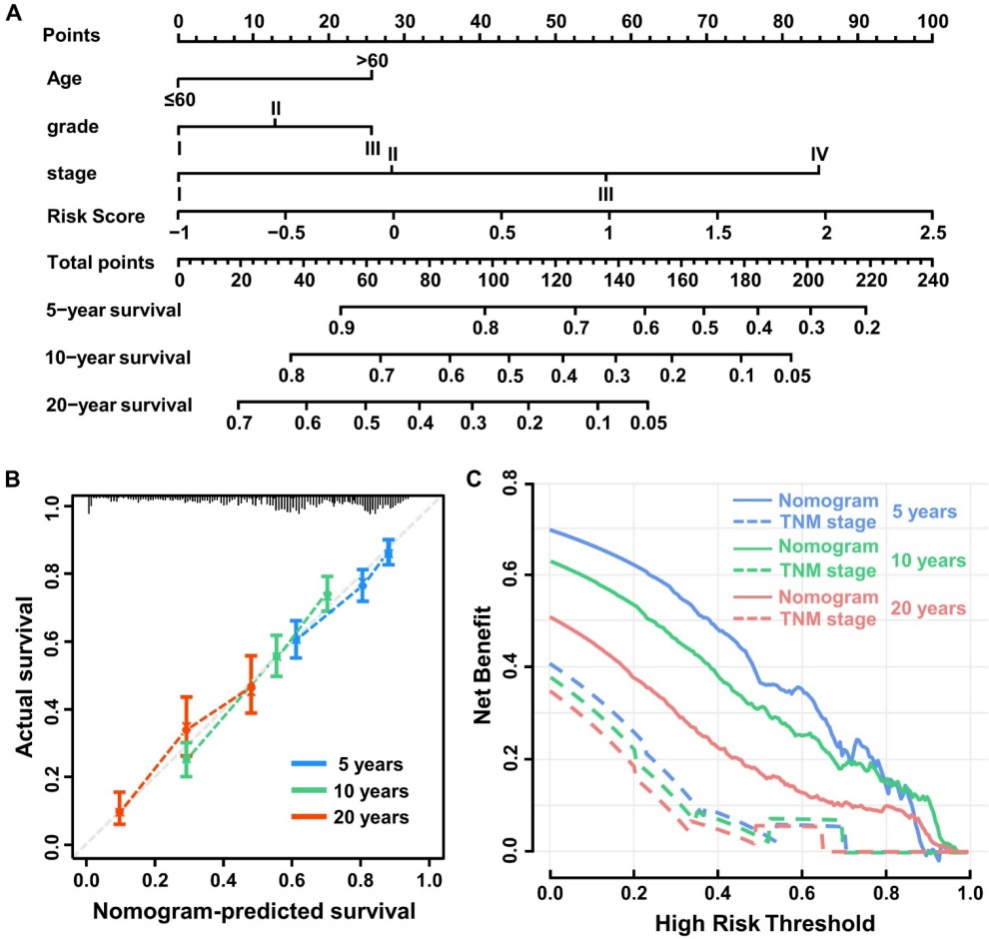
** Construction of the nomogram system. (A)** Nomogram predicting 5-, 10-, and 20-year overall survival for breast cancer patients in the training cohort based on immune score and other clinicopathological parameters. **(B)** The calibration curves of nomograms between predicted and observed 5-, 10- and 20-year OS in the training cohort. The dashed line of 45° represents the perfect prediction of the nomogram. **(C)** Decision curves of nomogram and TNM stage for 5-, 10- and 20-year outcome in the training cohort.

Univariable Cox regression analysis was performed to select independent clinicopathologically prognostic factors for OS, and the results showed significant relationships for age, tumor grade and tumor-node-metastasis (TNM) stage (Table 1). Subsequent multivariable Cox regression analysis showed that risk score, age, tumor grade, and TNM stage were independent prognostic factors for OS (Table 2). To create a quantitative method to predict OS, we integrated the immune score and independent clinicopathological prognostic factors, including age, tumor grade, and TNM stage, to construct a nomogram (Figure 4A).

To evaluate the predictive value of the nomogram, we compared Harrell's concordance index (C-index) of the nomogram with standard TNM staging in the training, validation, and test cohorts, and as shown in Table 3, the nomogram system improved the prognostic model of BC in all. Based on calibration plots, the predicted 5-, 10-, and 20-year survival probabilities of the nomogram performed well in both the training and validation cohorts (Figure 4B). Similarly, the decision curve showed better predictive accuracy than the standard TNM staging system (Figure 4C).

### The prognostic immune score model predicted the clinical characteristics of breast cancer patients

**Figure 5:**
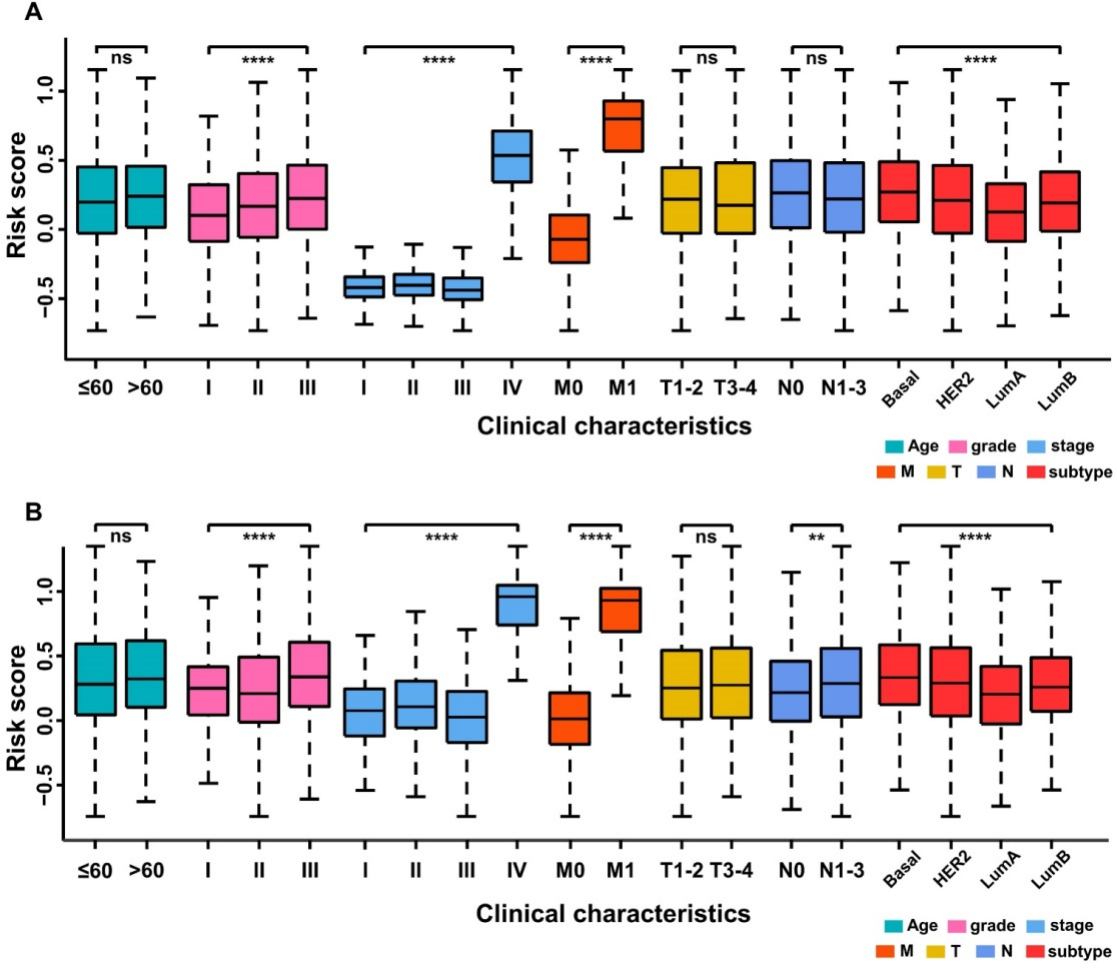
** Stratified analysis of clinical characteristics for the immune score of the immune prognostic model. (A)** Training cohort. **(B)** Validation cohort.

The relationship between the prognostic immune score and clinical characteristics was further investigated in the training and validation cohorts. In the former, the grade level (*p <* 0.0001), TNM stage (*p <* 0.0001), M category (*p <* 0.0001) and molecular subtype of BC (*p <* 0.0001) were significantly related to immune score, whereas age, T category and N category were not (Figure 5A). In the validation cohort (Figure 5B), a high immune score was positively associated with tumor grade (*p <* 0.0001), TNM stage (*p <* 0.0001), M category (*p <* 0.0001), N category (*p* < 0.01) and molecular subtype of BC (*p <* 0.0001).

### The prognostic immune score model predicted differential expression of genes involved in T-cell signal transduction, immune checkpoint, inflammation and EMT

**Figure 6:**
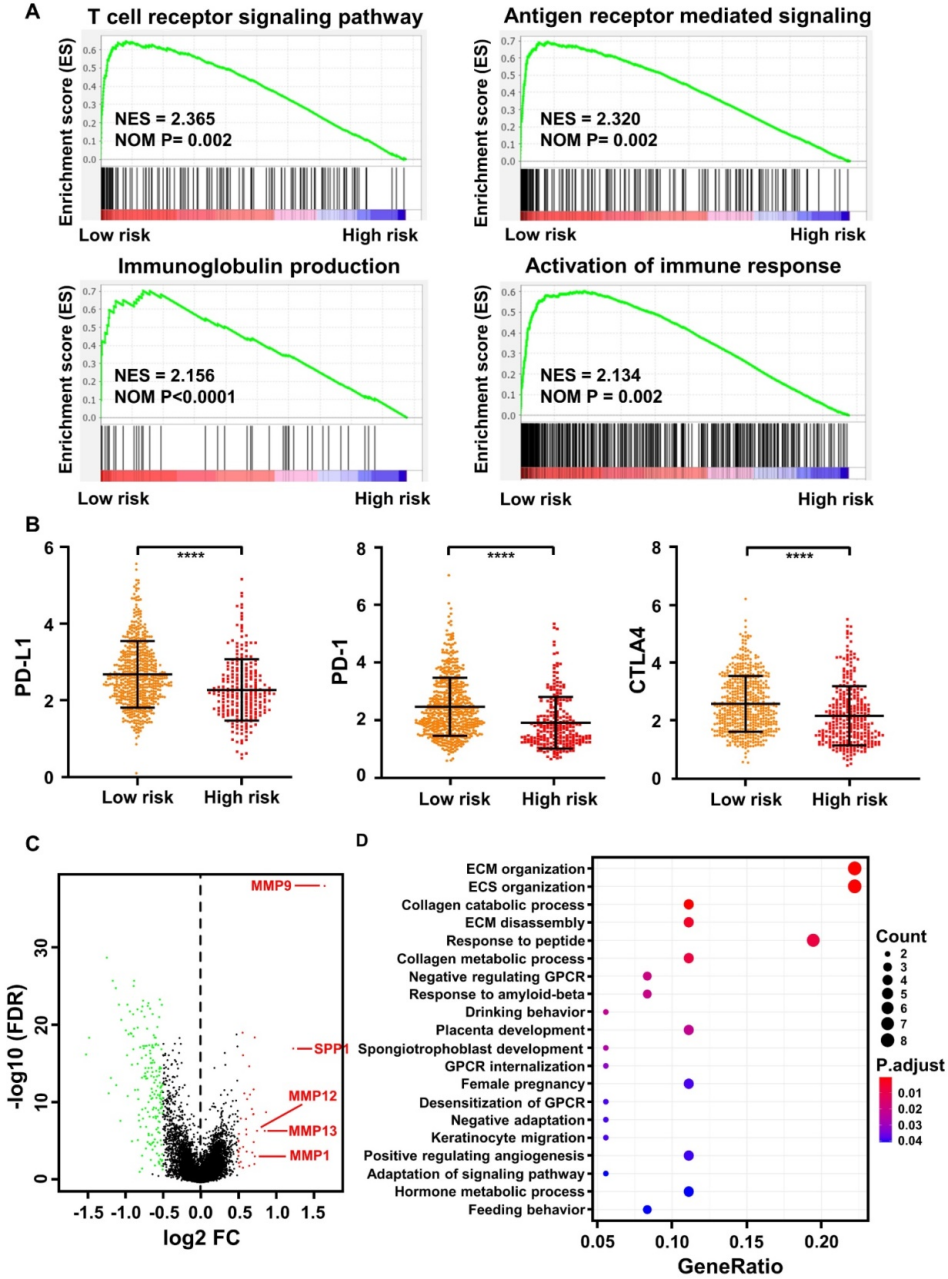
** Bioinformatics analysis of the characteristics and signaling pathways among patients in different risk groups. (A)** Gene set enrichment analysis (GSEA) for biological pathways and processes correlated with immune score values in the cohort from TCGA. NES, normalized enrichment score; NOM p: Nominal *p*-value. **(B)** PD-1, PD-L1, and CTLA4 mRNA expression between the low- and high-risk groups in the cohort from TCGA. **(C)** Volcano plot showing differentially expressed genes between the low- and high-risk groups in the cohort from TCGA. Genes labeled in red or green are significantly differentially up- or downregulated, respectively. FC: fold change; FDR: false discovery rate.** (D)** Gene Ontology analysis of the differentially expressed genes (DEGs). ECM: extracellular matrix; ECS: extracellular structure. GPCR: G Protein-Coupled Receptors.

The immune score of 836 TCGA samples was determined using the prognostic immune formula. All the samples were classified into low- and high-risk groups using the cutoff of 0.374. Gene-set enrichment analysis (GSEA) indicated that the low-risk group was highly enriched in activation of the T cell receptor signaling pathway, antigen receptor-mediated signaling pathway, immunoglobulin production, and activation of the immune response (Figure 6A).

Immune checkpoint blockade with immunotherapies, such as CTLA-4, PD-1, and PD-L1, has been proposed to be a promising approach to treat a variety of malignancies 38. Thus, we determined the expression level of several key immune checkpoint regulators as well as inflammatory mediators. As presented in Figure 6B, CTLA-4, PD-1, and PD-L1 expression was significantly higher in the low-risk group (*p <* 0.0001). In addition, other important immunomodulators or inflammatory mediators were increased in the low-risk group, including LAG3 (*p <* 0.0001), IL12A (*p <* 0.01), IL12B (*p <* 0.0001), IL6 (*p <* 0.05), IFNG (*p <* 0.0001), IDO1 (*p <* 0.0001), GZMB (*p <* 0.0001), and CD47 (*p <* 0.01) (Figure S5).

As a significant correlation between the M stage and immune score was observed in the training (Figure 5A) and validation (Figure 5B) cohorts, we further analyzed differentially expressed genes (DEGs) between the low- and high-risk groups in the cohort from TCGA. A total of 218 DEGs (38 upregulated and 180 downregulated genes, FDR *p*-value <0.05, Table S4) were identified in the high-risk group compared with the low-risk group. Among them, epithelial-mesenchymal transformation (EMT) markers such as MMP9, SPP1, MMP12, MMP13, and MMP1 were significantly overexpressed in the high-risk group (FDR *p-value <* 0.05, log FC>0.5, Figure 6C). Furthermore, according to Gene Ontology (GO) enrichment analysis, the genes in the high-risk group are mainly involved in extracellular matrix organization, extracellular structure organization, collagen catabolic process, collagen metabolic process, and extracellular matrix disassembly (Figure 6D).

## Discussion

The immune environment that surrounds cancer tissues can detect these tissues and inhibit their growth 30. In BC in particular, it has been reported that high levels of immune infiltration are associated with good clinical outcomes 4. In this study, we used CIBERSORT, which uses algorithm that well accommodates a large number of tumor samples that have been profiled by RNA sequencing, to estimate the proportion of immune cells in BC. This approach provides an alternative to flow or mass cytometry-based methods, and the cumbersome techniques of immunostaining are circumvented. CIBERSORT can also utilize archived RNA and cellular samples 27. Previous studies have validated the efficacy of the CIBERSORT technique in identifying a specific immune subset, which is a vast improvement over other techniques with very limited abilities 31-33. Additionally, LASSO regression was applied to construct an immune cell infiltration score model, a model capable of predicting near accurate survival times, as used in previous studies 34, 35. This immune score model is a novel prognostic tool designed to improve survival prediction after BC diagnosis. In this study, the immune score model was based on 22 immune cells, of which 12 showed a significant hazard ratio.

Moreover, the prognostic value of the immune score model was confirmed in training and validation cohorts. Our results showed a distinct separation of OS curves between patients who had high and low immune scores. In addition, the immune score was able to predict survival in the groups of patients, similar to TNM staging, indicating that such a model can be used for prognosis and may complement the existing TNM staging method.

Sufficient correlation between the immune score and expression of known inflammatory mediators such as PD-L1, CTLA-4, and LAG3 further supports its potential value 36, 37. The survival probability in the training, validation and test cohorts revealed significantly decreased survival. It has been observed that the immune score's predictive value may be suitable for large-scale data, and statistical significance was observed when the validation and test cohorts were combined.

According to the nomogram that included the immune score with TNM stage, a significant prognostic value was obtained by the combination compared to TNM stage alone. This is an indication that for prognosis, the immune score might be used to reinforce the prognostic ability of the TNM method. Indeed, the immune score value was verified in the nonoverlapping, validation cohort and in the test cohort, an indication of its utility in BC.

Adjuvant or neo-ACT is now regarded as the gold standard for the treatment of patients with stage II or III BC 38, 39. Nonetheless, candidates still face the challenge as to the selection criteria that are likely to be beneficial, and this remains a controversy. Many studies have assessed the connection between TILs and how efficient they are with ACT 40-42. Another study emphasized that high infiltration of immune cells contributes to an increased response to neo-ACT and ACT, and the use of chemotherapy to stimulate an anticancer immune response has been reported 43. Recently, a clinical trial found that the induction of chemotherapy in TNBC causes a favorable tumor immunologic microenvironment and increases sensitivity to PD-1 blockade 44. In another study by Wesolowski et al., the authors concluded that neo-ACT influences the immune microenvironment by downregulating CD4+ and upregulating CD8+ cells, which leads to a reduction in the number of TILs and CD8+ T cells in breast cancer samples 45. These reports are consistent with our present observations, as we observed a statistically significant potential link with chemotherapy for the METABRIC cohort. In contrast, no statistical significance was detected for the test cohort, which may be due to the small sample size as well as the difficulty in setting an optimum cutoff value. Previous studies have reported that chemotherapy sensitivity may be related to levels of lymphocyte infiltration into the tumor 46, 47. A possible mechanism involves the secretion of interferons by lymphocytes, which can sensitize cells to chemotherapy 48. In this study, levels of inflammatory mediators such as CTLA-4, PD-1, and PD-L1 were significantly higher in the lower-risk group. Some studies have, however, reported contrary results, whereby an increase in CTLA-4, PD-1, and PD-L1 has been associated with worse outcomes in cancer 49, 50. It is therefore important to verify this observation in a much broader database and in clinical samples. A similar trend for other critical immunomodulators or inflammatory mediators, such as LAG3, IL12A, IL12B, IL6, IFNG, IFNA1, IFNA2, IDO1, GZMB and CD47, was observed, with significantly higher expression in the low-risk group. This finding supports that interferon secretion might participate in the biological process of chemotherapy sensitization in BC patients with a low immune score. Innate and adaptive immunity may also be activated by immunogenic tumor cell death 51.

Although this study provides important evidence on the use of the immune score model in the prognosis of BC, it has some shortcomings. The study relied on retrospective data and might have missed some important information for each patient. For instance, anti-inflammatory drug use and the possible presence of any immune disorder that has a significant effect on the progression of the disease might have been examined, which hitherto have been excluded 52, 53. There is also difficulty in using a standardized cutoff for interpreting immune infiltration. Another important shortcoming is that the test cohort involved primary tumor tissues, whereas a good proportion of patients from publicly available gene expression datasets are analyzed with regard to metastatic sites, introducing some level of heterogeneity in the data and affecting the applicability of the nomogram in clinical practice. When considering the role of lymph nodes in the metastasis of BC, there is a need to consider the margin of a possible invasion of cancer in analyses. The gene expression profiles utilized were derived from a sample of the tumor tissues, with an associated impossibility of accounting for the location of immune cells, which should be considered in the model of the immune score. More data need to be collected prospectively to further validate these outcomes. Nevertheless, understanding the tumor immune microenvironment using the immune score provides important insight that will improve the diagnosis and prognosis of patients with BC.

## Methods

### Study population and gene expression profiling

The specimens for the test cohort were collected with the approval of hospitals in China. A total of 183 patients with BC who underwent synchronous neoadjuvant radiotherapy and chemotherapy (anthracycline + cyclophosphamide followed by taxane, AC-T) followed by mastectomy between 2002 and 2012 were included based on the following criteria: (1) pathology confirmed as primary BC following surgery; (2) complete clinical records and follow-up information available; (3) no history of other tumors; and (4) written informed consent. The exclusion criteria were insufficient breast tissue and insufficient clinical data regarding outcomes. We used the International Union against Cancer TNM classification system (5^th^ and 6^th^ editions) to classify resected tissues 54. Histological grades were classified as well-differentiated, moderately differentiated, and poorly differentiated. Clinical data were used for analysis based on ER, PR, HER2, and Ki67 expression levels. In our case, the staff members processing the clinical data were blinded to the details of the study. All patients provided written informed consent before enrolling in the study. The follow-up end date was September 30^th^, 2018, and the median follow-up time was 31 months.

A total of 130 core biopsy specimens and 53 surgical samples were immersed into RNAlater^TM^ solution (Qiagen, Germantown, MD, USA) and stored at -80 ºC until further analysis.

### Estimation of immune cell type fractions

Processed gene expression data were downloaded from public databases or obtained from raw files using the MAS5.0 algorithm and normalized using the limma package in R software (version 3.5.2) 55. To quantify the abundance of 22 TIICs in BC specimens, we applied CIBERSORT, an analytical tool, to provide an estimation of the proportions of cell types in a mixed cell population using normalized data 27. To run CIBERSORT, the following packages are required in R software: “e1071”, “parallel”, and “preprocessCore”. A file called “LM22.txt”, which contains a “signature matrix” of 547 genes, in R (obtained under Menu > Download from CIBERSORT web: https://cibersort.stanford.edu/download.php) is also required 56. The 22 types of infiltrating immune cells inferred by CIBERSORT include B cells, T cells, natural killer cells, macrophages, dendritic cells, eosinophils, and neutrophils. CIBERSORT derives a *p*-value for the deconvolution of each sample using Monte Carlo sampling, providing a measure of confidence in the results. At a threshold of *p <* 0.05, 5,038 samples of the inferred fractions of immune cell populations produced by CIBERSORT were considered accurate 10. The proportions of immune cells were predicted in each dataset separately.

### Sampling method

To improve the precision and accuracy of the prognostic model, the 5,038 samples were separated into training and validation sets in a ratio of 7:3 using stratified random sampling. Important clinical covariates, including age, molecular subtype, grade, TNM stage, and survival status, were taken into consideration to ensure equal distributions in the training and validation sets.

### GSEA

The transcriptome data of 836 BCs from TCGA were selected for GSEA analysis. For the cohort from TCGA, GSEA 4.0.3 software (downloaded from https://www.gsea-msigdb.org/gsea/downlodas.jsp) was used to identify GO terms enriched between the low- and high-risk groups in the c5 GO database (c5.all.v6.2.symbols). The significance threshold was set at *p* < 0.05.

### Statistical analysis

The Mann-Whitney U test was utilized to compare two groups and the Kruskal-Wallis test to compare multiple groups. Univariate, LASSO, and multivariate Cox regression analyses were applied to identify the most significant immune cells to build a prognostic model. Immune cells were considered significant when the *p*-value was <0.05 in univariate Cox regression analysis. Subsequently, we used LASSO-penalized Cox regression to filter out less relevant factors. Finally, multivariate Cox regression analysis was applied to optimize the model. The optimal cutoff values were calculated based on the association between OS and cell fraction in the training cohort using the survminer package in R. Kaplan-Meier analysis and the log-rank test were employed to evaluate correlations between the proportion of immune cells and OS. The prognostic value of the nomogram for 5, 10, and 20 years was evaluated by the c-index 57. Results with two-sided *p-*values of <0.05 were considered to be statistically significant. The statistical analyses were conducted using SPSS version 25 (IBM, New York, USA) and R software (3.5.2).

## Supplementary Material

Supplementary figures and tables.

Supplementary table S1.

Supplementary table S4.

## Figures and Tables

**Table 1:** Results of Univariable Cox regression analysis

Univariable Cox Regression Analysis				
Variables	Training cohort		Validation & Test cohort	
	HR (95%CI)	*P*-value	HR (95%CI)	*P*-value
Risk score*	2.72 (2.40-3.08)	**<0.0001**	2.10 (1.74-2.53)	**<0.0001**
Age (>60 vs ≤60)	1.71 (1.51-1.95)	**<0.0001**	1.45 (1.19-1.76)	**0.0002**
Grade (High vs Low)	1.67 (1.45-1.92)	**<0.0001**	1.69 (1.36-2.08)	**<0.0001**
**Stage (vs stage I)**				
II	1.38 (1.13-1.68)	**0.001**	2.16 (1.51-3.08)	**<0.0001**
III	2.47 (1.94-3.15)	**<0.0001**	3.24 (2.18-4.80)	**<0.0001**
IV	6.83 (5.30-8.81)	**<0.0001**	8.69 (5.70-13.24)	**<0.0001**
**Subtype (vs basal-like)**				
HER2	1.22 (1.02-1.46)	**0.03**	1.25 (0.95-1.63)	0.11
Lum A	0.55 (0.46-0.66)	**<0.0001**	0.71 (0.54-0.93)	**0.01**
Lum B	0.99 (0.84-1.16)	0.86	0.99 (0.77-1.26)	0.93
Normal like	1.13 (0.85-1.50)	0.39	0.93 (0.60-1.44)	0.74

*Continuous variable.

**Table 2:** Results of Multivariable Cox regression analysis

Multivariable Cox Regression Analysis				
Variables	Training cohort		Validation & Test cohort	
	HR (95%CI)	*P*-value	HR (95%CI)	*P*-value
Risk score*	1.62 (1.24-2.11)	**0.0004**	1.40 (1.02-1.91)	**0.04**
Age (≥60 vs <60)	1.49 (1.24-1.80)	**<0.0001**	1.82 (1.35-2.46)	**0.0001**
Grade (High vs Low)	1.30 (1.06-1.60)	**0.01**	1.43 (1.04-1.98)	**0.03**
**Stage (vs stage I)**				
II	1.27 (1.02-1.60)	**0.03**	1.98 (1.31-2.98)	**0.001**
III	2.86 (2.06-3.98)	**<0.0001**	2.80 (1.66-4.69)	**0.0001**
IV	3.40 (2.26-5.12)	** <0.0001**	5.25 (2.76-9.98)	**<0.0001**
**Subtype (vs basal-like)**				
HER2	1.31 (0.99-1.74)	0.05	1.44 (0.97-2.14)	0.07
LumA	0.97 (0.73-1.30)	0.86	1.40 (0.88-2.23)	0.15
LumB	1.14 (0.88-1.49)	0.32	1.17 (0.79-1.73)	0.44
Normal like	1.19 (0.77-1.83)	0.43	1.17 (0.64-2.14)	0.60

*Continuous variable.

**Table 3:** Harrell's concordance indexes of TNM stage and nomogram system

Cohort	C-index (95%CI)	
	TNM stage	Nomogram
Training	0.630 (0.619-0.640)	0.665 (0.653-0.677)
Validation	0.674 (0.657-0.691)	0.691 (0.663-0.719)
Test	0.512 (0.466-0.557)	0.885 (0.823-0.947)
