# Supplementary material for: An immune cell infiltration-based immune score model predicts prognosis and chemotherapy effects in breast cancer
Source: Theranostics. 2020 Oct 25;10(26):11938–49. doi: 10.7150/thno.49451 (PMC7667685; doi:10.7150/thno.49451)
Supplement: Supplementary file 1 — Supplementary figures and tables. [file thnov10p11938s1.pdf]

Supplementary data：

1. Supplementary figures and tables:

Fig. S1-S2. Kaplan-Meier curves of overall survival in patients with different molecular types of breast cancer in the training cohort (Fig. S1A-D) and validation cohort (Fig. S2A-D).

Fig. S3. Subgroup analysis of adjuvant chemotherapy (ACT) benefit for DFS of low- and high-risk patients in the METABRIC database.

Fig. S4. The immune risk score values from the pCR and nonpCR groups in the test cohort. Violin plot showing immune score values between the pCR and nonpCR groups in the test cohort. pCR, pathologic complete response.

Fig. S5. Evaluation of expression of immune checkpoint genes (LAG3, IL12A, IL12B, IL6, IFNG, IDO1, GZMB, CD47) between low- and high-risk groups in the cohort from TCGA.

Fig.S1

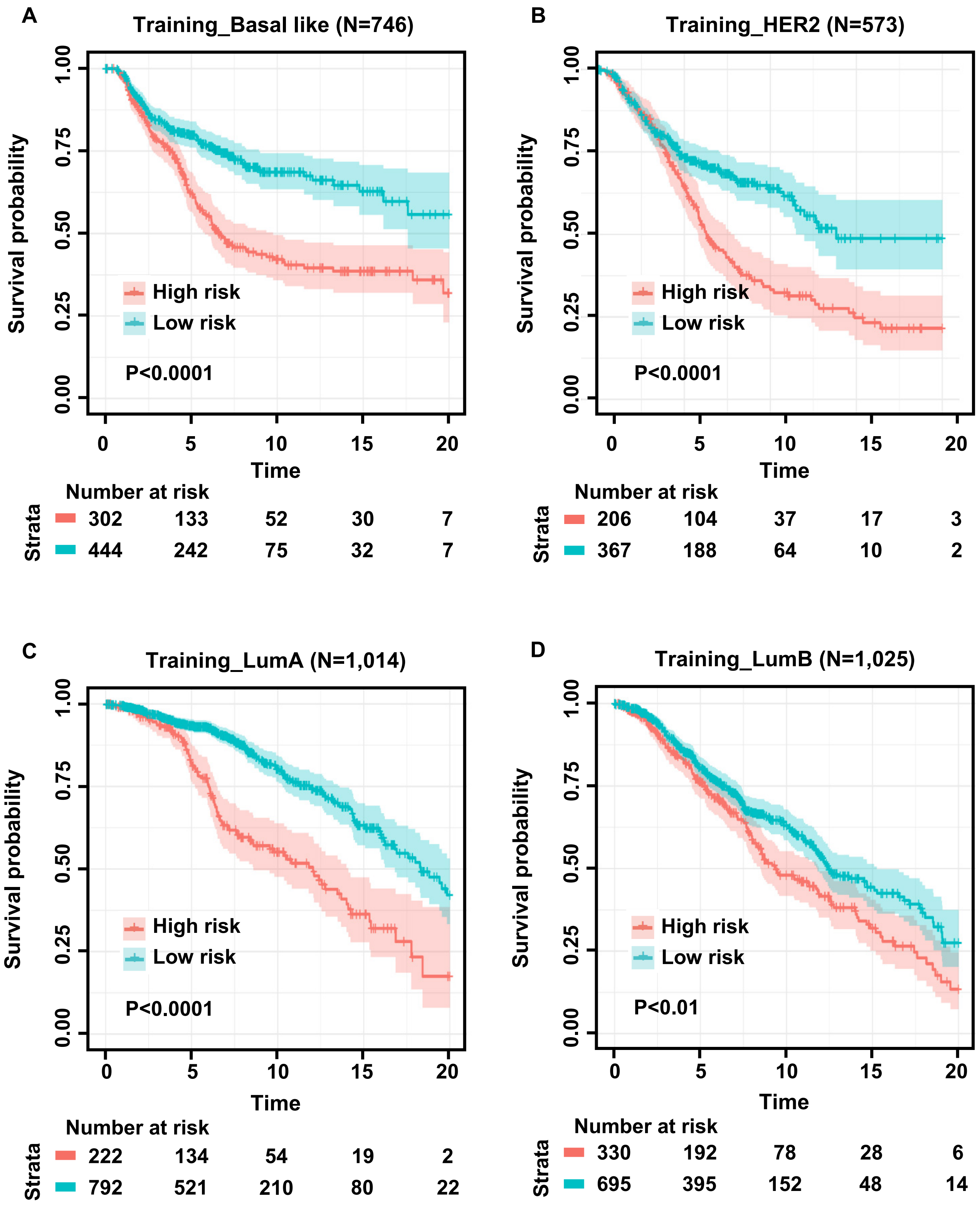
A
Training_Basal like (N=746)
Survival probability
1.00
0.75
0.50
0.25
0.00
High risk
Low risk
P<0.0001
0
5
10
15
20
Time
Number at risk
Strata
302 133 52 30 7
444 242 75 32 7
B
Training_HER2 (N=573)
Survival probability
High risk
Low risk
P<0.0001
Time
Number at risk
Strata
206 104 37 17 3
367 188 64 10 2
C
Training_LumA (N=1,014)
Survival probability
High risk
Low risk
P<0.0001
Time
Number at risk
Strata
222 134 54 19 2
792 521 210 80 22
D
Training_LumB (N=1,025)
Survival probability
High risk
Low risk
P<0.01
Time
Number at risk
Strata
330 192 78 28 6
695 395 152 48 14

Fig.S2

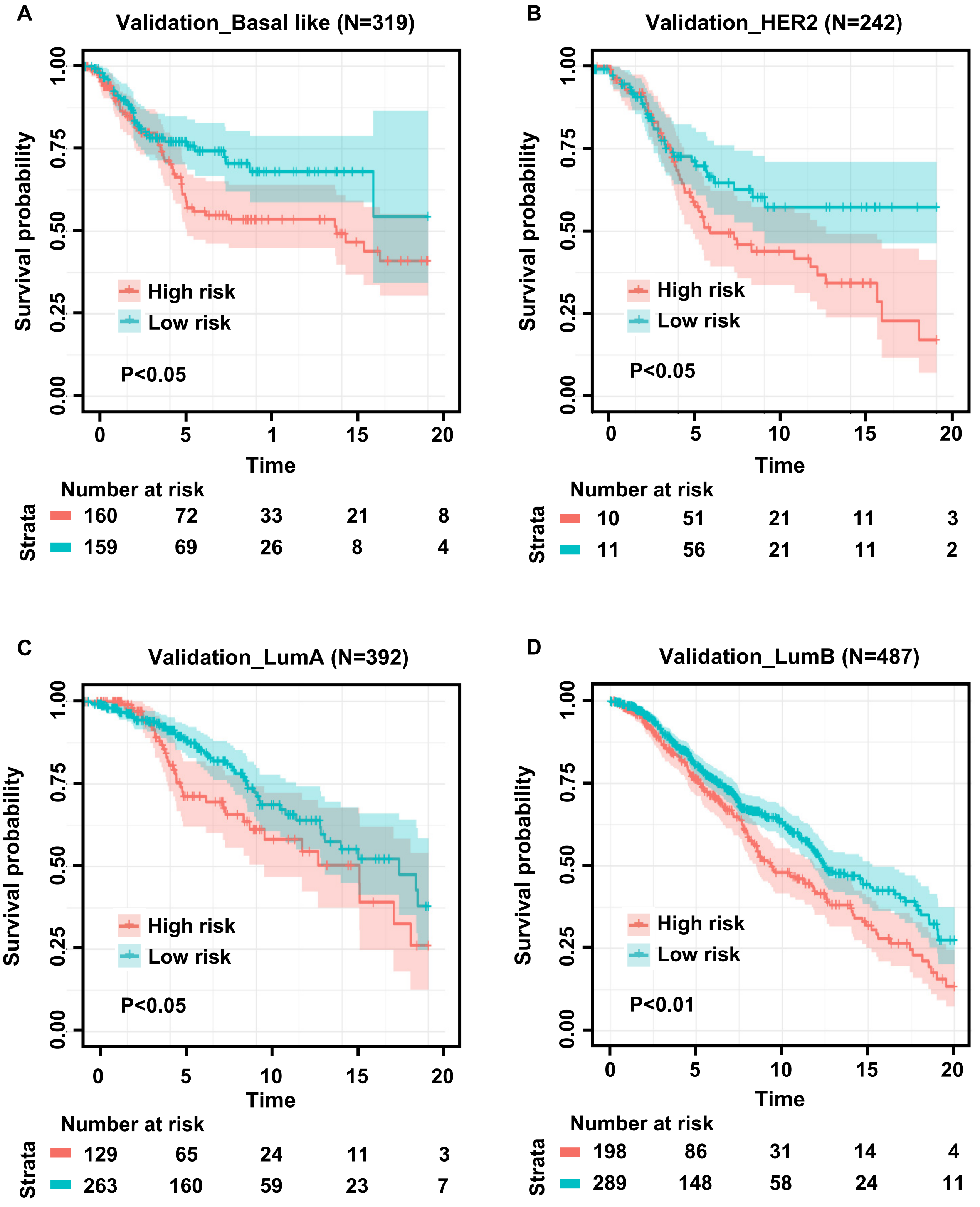
A
Validation_Basal like (N=319)
Survival probability
1.00
0.75
0.50
0.25
0.00
High risk
Low risk
P<0.05
0
5
1
15
20
Time
Number at risk
Strata
160 72 33 21 8
159 69 26 8 4
B
Validation_HER2 (N=242)
Survival probability
1.00
0.75
0.50
0.25
0.00
High risk
Low risk
P<0.05
0
5
10
15
20
Time
Number at risk
Strata
10 51 21 11 3
11 56 21 11 2
C
Validation_LumA (N=392)
Survival probability
1.00
0.75
0.50
0.25
0.00
High risk
Low risk
P<0.05
0
5
10
15
20
Time
Number at risk
Strata
129 65 24 11 3
263 160 59 23 7
D
Validation_LumB (N=487)
Survival probability
1.00
0.75
0.50
0.25
0.00
High risk
Low risk
P<0.01
0
5
10
15
20
Time
Number at risk
Strata
198 86 31 14 4
289 148 58 24 11

Fig.S3

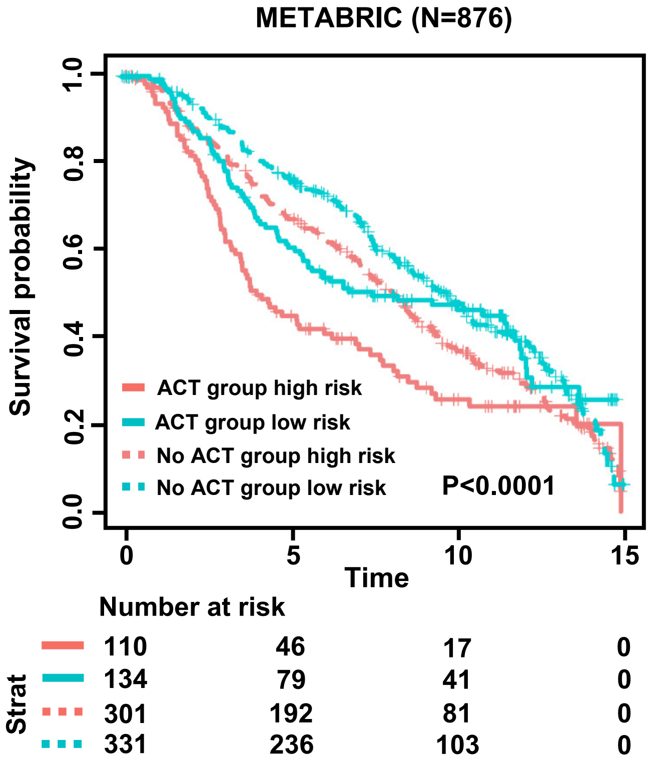
METABRIC (N=876)
Survival probability
1.0
0.8
0.6
0.4
0.2
0.0
ACT group high risk
ACT group low risk
No ACT group high risk
No ACT group low risk
P<0.0001
0
5
10
15
Time
Number at risk
Strat
110 46 17 0
134 79 41 0
301 192 81 0
331 236 103 0

Fig.S4

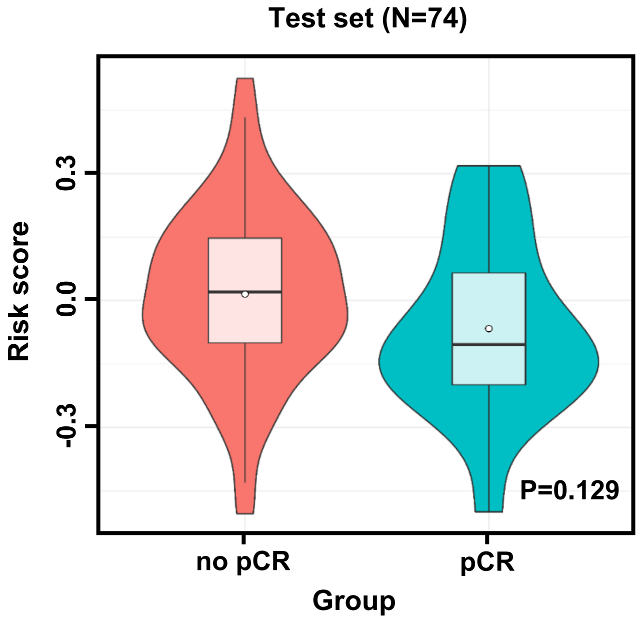
Test set (N=74)
Risk score
0.3
0.0
-0.3
P=0.129
no pCR
pCR
Group

26 **Fig.S5**

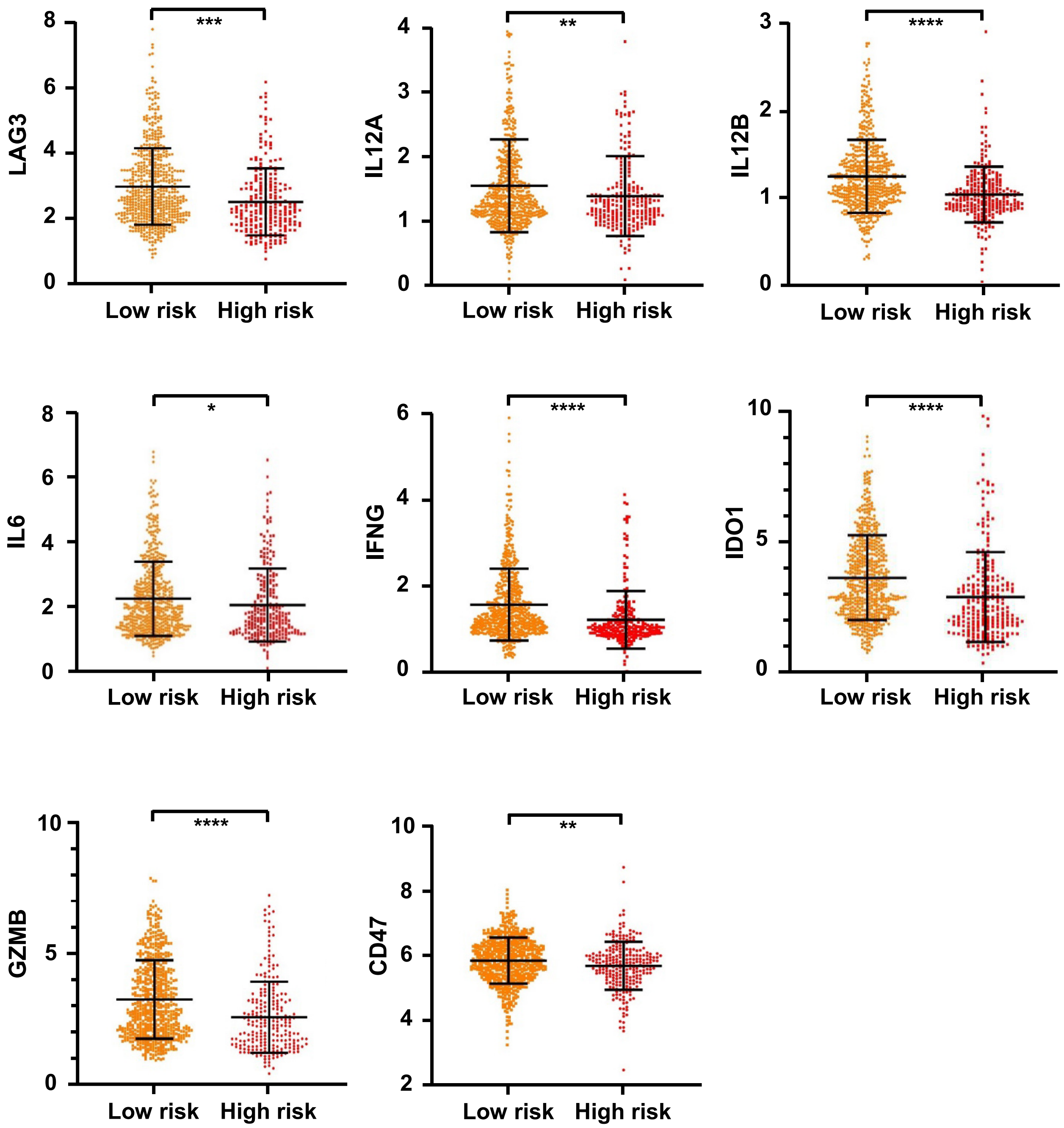

Table S1. Summary of included studies (Excel).

Table S2. Baseline patient characteristics

Characteristics	Number of patients (%)		
	Training set (N=3526)	Validation set (N=1512)	Test set (N=74)
**Age (years)**			
≤ 60	1788 (50.7%)	762 (50.4%)	68 (91.9%)
> 60	1033 (29.3%)	446 (29.5%)	6 (8.1%)
**Tumor grade**			
Low grade	881 (25.0%)	374 (24.7%)	23 (31.1%)
High grade	1125 (31.9%)	480 (31.7%)	43 (58.1%)
**PAM50 subtype**			
Basal like	746 (21.1%)	319 (21.1%)	25 (33.8%)
HER2	573 (16.3%)	242 (16.0%)	35 (47.3%)
Luminal like	2038 (57.8%)	879 (58.1%)	8 (10.8%)
Normal like	169 (4.8%)	69 (4.6%)	6 (8.1%)
**Stage**			
Early	1583 (44.9%)	679 (44.9%)	7 (9.5%)
Advanced	479 (13.6%)	207 (13.7%)	67 (90.5%)

Table S3. Coefficients of immune markers for predicting breast cancer prognosis.

Immune cell marker	Coefficients	SE	*P*-Value
T cells CD4 memory resting	-1.761	0.490	0.0003
T cells regulatory (Tregs)	2.526	0.629	<0.0001
T cells gamma delta	-2.334	0.662	0.0004
NK cells activated	3.408	0.764	<0.0001
Monocytes	2.645	0.908	0.004
Macrophages M0	1.591	0.274	<0.0001
